# Ferroptotic damage promotes pancreatic tumorigenesis through a TMEM173/STING-dependent DNA sensor pathway

**DOI:** 10.1038/s41467-020-20154-8

**Published:** 2020-12-11

**Authors:** Enyong Dai, Leng Han, Jiao Liu, Yangchun Xie, Herbert J. Zeh, Rui Kang, Lulu Bai, Daolin Tang

**Affiliations:** 1 Department of Oncology and Hematology, China-Japan Union Hospital of Jilin University, Changchun, Jilin 130031 China; 2 Protein Modification and Degradation Lab, Third Affiliated Hospital of Guangzhou Medical University, Guangzhou, Guangdong 510120 China; 3 Department of Oncology, The Second Xiangya Hospital, Central South University, Changsha, Hunan 410008 China; 4 Department of Surgery, UT Southwestern Medical Center, Dallas, TX 75390 USA; 5 Department of Pediatric Hematology, First Hospital of Jilin University, Changchun, Jilin 130021 China

**Keywords:** Cancer microenvironment, Gastrointestinal cancer

## Abstract

Ferroptosis is a more recently recognized form of cell death that relies on iron-mediated oxidative damage. Here, we evaluate the impact of high-iron diets or depletion of *Gpx4*, an antioxidant enzyme reported as an important ferroptosis suppressor, in the pancreas of mice with cerulean- or L-arginine-induced pancreatitis, and in an oncogenic *Kras* murine model of spontaneous pancreatic ductal adenocarcinoma (PDAC). We find that either high-iron diets or *Gpx4* depletion promotes 8-OHG release and thus activates the TMEM173/STING-dependent DNA sensor pathway, which results in macrophage infiltration and activation during *Kras*-driven PDAC in mice. Consequently, the administration of liproxstatin-1 (a ferroptosis inhibitor), clophosome-mediated macrophage depletion, or pharmacological and genetic inhibition of the 8-OHG-TMEM173 pathway suppresses *Kras*-driven pancreatic tumorigenesis in mice. GPX4 is also a prognostic marker in patients with PDAC. These findings provide pathological and mechanistic insights into ferroptotic damage in PDAC tumorigenesis in mice.

## Abstract (web summary)

Ferroptosis is an iron-dependent mechanism of cell death. In this mouse study, the authors show that diets high in iron or depletion of the antioxidant Gpx4 potentiates pancreatic damage and tumour formation by activating the DNA damage pathway and recruiting macrophages to the pancreas.

## Introduction

Pancreatic cancer remains one of the challenges in medicine and accounts for ~3% of all cancers in the United States and ~7% of all cancer deaths. Pancreatic ductal adenocarcinoma (PDAC), a type of exocrine tumor, is the most common pathological type of pancreatic cancer^1^. Although many factors contribute to the transition of a normal pancreatic duct to a pre-invasive precursor lesion into invasive PDAC, this progression is mainly driven by intrinsic *Kras* mutations within an inflammatory tumor microenvironment^2^. Understanding the components and function of the pancreatic tumor microenvironment may lead to developing new diagnostic and better therapeutic approaches^3^.

Impaired cell death is now recognized as an early step toward the development of various cancers, including PDAC^4,5^. Ferroptosis was initially described as a form of *Ras* mutation-dependent regulated cell death characterized by excessive iron-induced oxidative damage^6,7^, but subsequent analysis indicates that it occurs in both *Ras* mutation-dependent and *Ras* mutation-independent pathways^8,9^. Glutathione peroxidase 4 (GPX4), a phospholipid hydroperoxidase, appears to play a major role in protecting against ferroptosis by the detoxification of oxidative damage to membrane lipids^10^. The conditional depletion of *Gpx4* in certain tissues (e.g., kidney) triggers lipid peroxidation-dependent ferroptotic injury in mice^11^. The modulation of ferroptosis may have therapeutic potential in iron overload-associated diseases, including cancer and neurological disorders^12,13^. Although the pharmacological induction of ferroptosis is becoming a potential anticancer strategy^14,15^, the functional relevance of ferroptotic damage in oncogenic progression is poorly understood.

Here, we investigate the pathologic role of iron and GPX4 in *Kras*-driven PDAC, using pancreatic *Gpx4* conditional knockout (KO) mice or high-iron diet models. We demonstrate that oxidized nucleobase release from high-iron diets or *Gpx4* depletion induces the activation of a transmembrane protein 173 (TMEM173, also known as STING)-dependent DNA sensor pathway that promotes macrophage infiltration and activation, and thereby enables pancreatic cancer initiation.

## Results

### High-iron diets or *Gpx4* depletion promotes experimental pancreatitis

Because pancreatitis is an established risk factor for the development of pancreatic cancer^1^, we first investigated the impact of ferroptosis on two experimental models of acute pancreatitis induced by cerulean (an analog of cholecystokinin) or l-arginine (a conditionally essential amino acid). After 3 months on the high-iron diet, mice had more pancreatic iron (662.2 ± 51.05 µg/g) than control diet mice (59.62 ± 4.04 µg/g). The mice with high-iron diets were substantially more susceptible to cerulean- or l-arginine-induced pancreatitis with significantly higher mortality rates compared to control diet mice (Supplementary Fig. 1a). A histological assessment of pancreatic damage revealed exaggerated acinar cell death, leukocyte infiltration, and interstitial edema in the high-iron diet group (Supplementary Fig. 1b). Serum amylase (Supplementary Fig. 1c), pancreas trypsin activity (Supplementary Fig. 1d), and pancreas myeloperoxidase activity (Supplementary Fig. 1e) were significantly increased in the high-iron diet group, indicating that high iron levels accelerate pancreatitis progress.

**Fig. 1:**
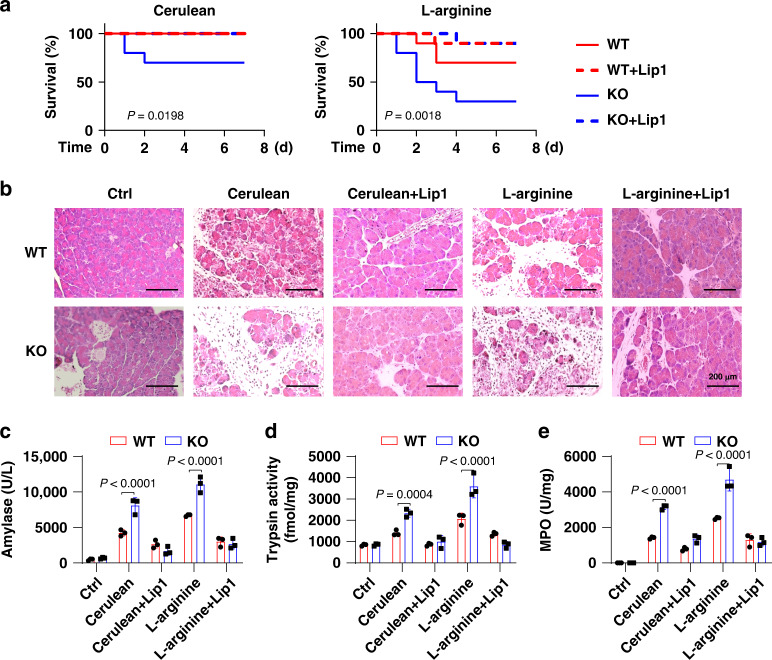
*Gpx4* depletion promotes experimental pancreatitis. **a** Survival of wild-type (WT) or *Pdx1-Cre;Gpx4*^*−/−*^ (KO) mice with cerulean- or l-arginine-induced pancreatitis with or without liproxstatin-1 (Lip1) treatment (*n* = 10 mice/group; one-sided log-rank [Mantel–Cox] test). **b**–**e** In parallel, pancreas histology (**b**), plasma amylase activity (**c**), pancreatic trypsin activity (**d**), and pancreatic myeloperoxidase (MPO) activity (**e**) at 24 h were assayed (*n* = 3 mice/group; two-way ANOVA with Tukey’s multiple comparisons test). Data in **c**–**e** are presented as mean ± SD. Data are from two or three independent experiments.

We next determined the effects of GPX4-mediated antioxidant response on experimental pancreatitis. The conditional KO of *Gpx4* in the pancreas of mice (*Pdx1-Cre;Gpx4*^*−/−*^, termed KO mice) did not affect pancreatic development (such as birth weight, exocrine function [e.g., blood amylase], and endocrine function [e.g., blood glucose]) (Supplementary Fig. 2) and iron level (64.72 ± 10.23 µg/g in WT versus 73.81 ± 17.31 µg/g in KO mice), but resulted in cerulean- or l-arginine-induced pancreatitis developing more rapidly than in wild-type (WT) mice, with increased mortality (Fig. 1a), pancreatic injury (Fig. 1b), and pancreatitis-associated enzymes, including amylase, trypsin, and myeloperoxidase (Fig. 1c–e).

In contrast, the administration of liproxstatin-1, a widely used ferroptosis inhibitor through its strong antioxidant activity^11^, protected against cerulean- or l-arginine-induced pancreatitis, especially in the presence of a high-iron diet or *Gpx4* depletion (Fig. 1a–e and Supplementary Fig. 1a–e). These findings suggest that iron overload and *Gpx4* depletion accelerates the progression of experimental pancreatitis via oxidative damage.

### High-iron diets or *Gpx4* depletion promotes *Kras*-driven pancreatic tumorigenesis

**Fig. 2:**
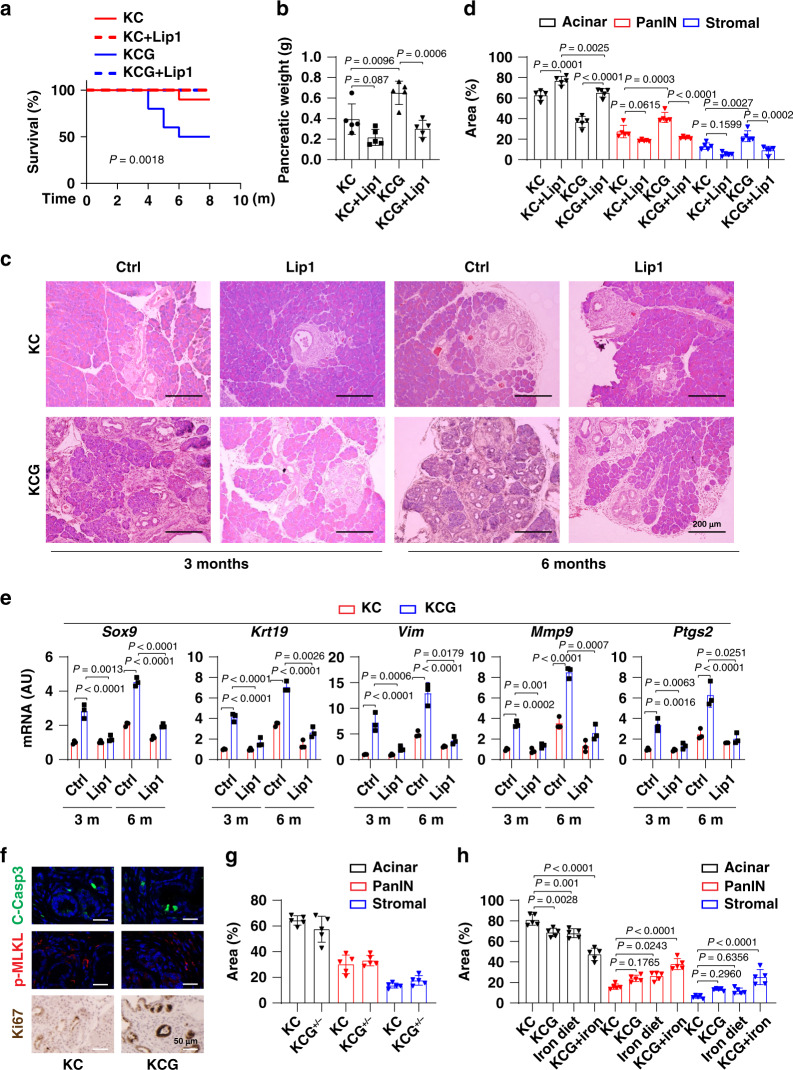
*Gpx4* depletion promotes *Kras*-driven pancreatic tumorigenesis. **a** Survival of *Pdx1-Cre;K-Ras*^*G12D/+*^ (KC) and *Pdx1-Cre;K-Ras*^*G12D/+*^*;Gpx4*^*−/−*^ (KCG) mice with or without liproxstatin-1 (Lip1) treatment (*n* = 10 mice/group; one-sided log-rank [Mantel–Cox] test). **b** Pancreatic weight of the indicated KC and KCG mice (6 months; *n* = 5 mice/group; two-way ANOVA with Tukey’s multiple comparisons test). **c** Representative pancreas histology of the indicated KC and KCG mice. **d** Percentages of histological structures in the pancreas of the indicated KC and KCG mice (6 months; *n* = 5 mice/group; two-way ANOVA with Tukey’s multiple comparisons test). **e**, **f** Relative gene or protein expression in the pancreas of the indicated KC and KCG mice (*n* = 3 mice/group; two-way ANOVA with Tukey’s multiple comparisons test). **g** Percentages of histological structures in the pancreas of the indicated KC and KCG^+/*−*^ mice (6 months; *n* = 5 mice/group; two-way ANOVA with Tukey’s multiple comparisons test). **h** Percentages of histological structures in the pancreas of the indicated KCG mice with or without high-iron diets (3 months; *n* = 5 mice/group; two-way ANOVA with Tukey’s multiple comparisons test). Data in **b**, **d**, **e**, **g**, and **h**) are presented as mean ± SD. Data are from two or three independent experiments.

The ability of mutant *Kras* to drive PDAC was not successfully investigated until the generation of mice with a *Cre*-inducible conditional allele (*Pdx1-Cre;Kras*^*G12D/+*^, termed KC mice) targeting the endogenous *Kras* locus^16^. Compared to KC mice, either *Gpx4* depletion (*Pdx1-Cre;Kras*^*G12D/+*^*;Gpx4*^*−/−*^, termed KCG mice) or a high-iron diet further increased *Kras*-mediated animal death (Fig. 2a and Supplementary Fig. 3a), increased pancreatic weight (Fig. 2b and Supplementary 3b), pancreatic intraepithelial neoplasia (PanIN) formation, and stromal response (Fig. 2c, d and Supplementary 3c, d). In contrast, the normal acinar cells were reduced by *Gpx4* depletion or a high-iron diet (Fig. 2c, d and Supplementary 3c, d). As expected, the pancreatic iron of KC mice (875.8 ± 46.5 µg/g) was higher than the control diet (174.7 ± 30.12 µg/g). There was no difference in pancreatic iron between KC and KCG mice on a normal diet. Increased tumor invasion or metastasis to the liver and lung was observed in KCG mice at age of 10–12 months (50% [5/10] in the KCG group versus 20% [2/10] in the KC group). These findings indicate that high-iron diets or *Gpx4* depletion accelerates *Kras*-mediated PDAC in the pancreas.

We next investigated the effects of *Gpx4* depletion or a high-iron diet on *Kras*-driven molecular events involved in acinar-to-ductal metaplasia, ductal lesions, stromal response, and metastasis. Indeed, the messenger RNA (mRNA) expression of molecular markers of acinar-to-ductal metaplasia (e.g., SRY-box 9 [*Sox9*]), ductal lesions (e.g., keratin 19 [*Krt19/Ck19*]), stromal response (e.g., vimentin [*Vim*]), and metastasis (e.g., matrix metallopeptidase 9 [*Mmp9*]) were upregulated by either *Gpx4* depletion or a high-iron diet (Fig. 2e and Supplementary 3e).

Conversely, the ferroptosis inhibitor liproxstatin-1 protected against *Kras*-driven animal death, and pathological and molecular changes in mice with *Gpx4* depletion (Fig. 2a–e) or a high-iron diet (Supplementary 3a–e). Moreover, the expression of prostaglandin-endoperoxide synthase 2 (PTGS2, a marker of ferroptosis, but not the driving factor of ferroptosis)^10^ and Ki67 (a marker of tumor cell proliferation), but not cleaved caspase-3 (a marker of apoptosis) and phosphorylated mixed lineage kinase domain-like pseudokinase (p-MLKL, a marker of necroptosis), in the pancreas was upregulated by either *Gpx4* depletion or a high-iron diet in *Kras*-driven pancreatic tumorigenesis (Fig. 2f and Supplementary 3f)*.* These animal studies indicate that ferroptotic damage may promote *Kras*-driven pancreatic tumorigenesis.

Notably, heterozygous lack of *Gpx4* (*Pdx1-Cre;Kras*^*G12D/+*^*;Gpx4*^*−/+*^, termed KCG^*−*/+^ mice) failed to accelerate *Kras*-mediated animal death (14.3% [1/7] in the KCG^*−*/+^ group versus 10% [1/10] in the KC group), PanIN formation, and stromal response (Fig. 2g), indicating that the complete loss of *Gpx4* is necessary to increase PDAC development. In addition, at 3 months of age, homozygous *Gpx4* depletion and a high-iron diet had a synergistic effect on *Kras*-mediated PanIN formation and stromal response (Fig. 2h). In vitro, celecoxib (PTGS2 inhibitor) or exogenous prostaglandin E2 (PGE2, an enzymatic product of PTGS2) failed to affect the growth of *Gpx4*^*−/−*^ PDAC cells, indicating that the PTGS2 pathway may not be important for tumor growth associated with GPX4 depletion (Supplementary 3g).

### Macrophage depletion decreases pancreatic tumorigenesis

Given that macrophages are major players in the early stages of pancreatic tumorigenesis^17,18^, we next tested the impact of *Gpx4* depletion or a high-iron diet on macrophage infiltration and activation in the tumor microenvironment. Macrophage infiltration was examined by immunofluorescence staining of F4/80. Tumor-associated macrophages (TAMs) were increased in *Kras-*driven mice with *Gpx4* depletion or a high-iron diet (Supplementary Fig. 4a), indicating that iron and GPX4 are regulators of macrophage infiltration in pancreatic tumor microenvironment.

Macrophages may polarize into either M1- or M2-like macrophages depending on the stage of tumor progression^19^. M1 macrophages are mainly abundant in chronic inflammatory sites where tumors are initiated and start to develop. During cancer progression, macrophages switch to an M2-like phenotype to express immunosuppressive signals^19^. In agreement with these notions, isolated TAMs from *Gpx4* depletion or a high-iron diet group exhibited higher mRNA expression of markers for M1-like (e.g., tumor necrosis factor [*Tnf*] and interleukin-6 [*Il6*]) at 3 months and M2-like (e.g., nitric oxide synthase 2 [*Nos2*/*iNos*] and arginase-1 [*Arg1*]) at 6 months (Supplementary Fig. 4b, c). The administration of liproxstatin-1 reduced macrophage infiltration (Supplementary Fig. 4a) in tumors, and M1 and M2 marker mRNA expression (especially *Il6* and *Nos2*) (Supplementary Fig. 4b, c) in TAMs, indicating that oxidative damage may promote TAM infiltration and activation. As expected, the increased expression of YM1 (M2 marker in mice^20^) in the tumor microenvironment of KCG and high-iron diet mice was decreased by liproxstatin-1 (Supplementary Fig. 3d).

**Fig. 3:**
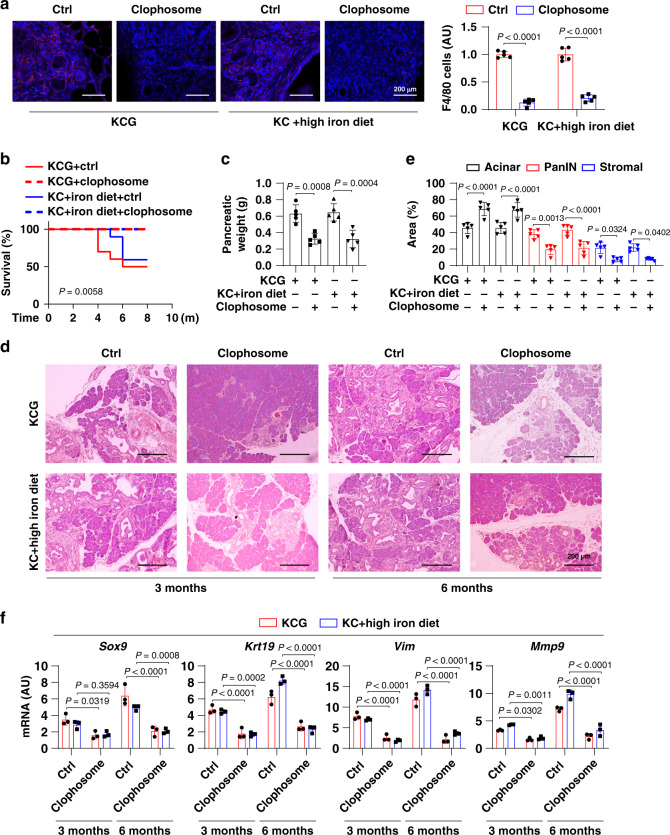
Macrophage depletion decreases pancreatic tumorigenesis. **a** Representative images of immunofluorescence staining of macrophages (red) in the pancreas in indicated mice at the age of 3 months after treatment with clophosome or control liposomes (Ctrl) (*n* = 5 mice/group; one-tailed *t* test). **b** Survival of *Pdx1-Cre;K-Ras*^*G12D/+*^*;Gpx4*^*−/−*^ (KCG) or *Pdx1-Cre;K-Ras*^*G12D/+*^ (KC) mice with high-iron diets after treatment with clophosome or control liposomes (Ctrl) (*n* = 10 mice/group; one-sided log-rank [Mantel–Cox] test). **c** Pancreatic weight of the indicated mice (6 months; *n* = 5 mice/group; one way ANOVA with Tukey’s multiple comparisons test). **d** Representative pancreas histology of the indicated mice. **e** Percentages of histological structures in the pancreas of the indicated mice (6 months; *n* = 5 mice/group; two-way ANOVA with Tukey’s multiple comparisons test). **f** Relative gene expression in the pancreas of the indicated mice (*n* = 3 mice/group; two-way ANOVA with Tukey’s multiple comparisons test). Data in **a**, **c**, **e**, and **f** are presented as mean ± SD. Data are from two or three independent experiments.

To further determine the role of macrophages in high-iron diet- or *Gpx4* depletion-dependent tumorigenesis, we used clophosome (clodronate liposomes^21^) to deplete macrophages (Fig. 3a). Compared to control liposomes, the pharmacological inhibition of macrophages by clophosome reduced animal death (Fig. 3b), pancreatic weight (Fig. 3c), PanIN formation, and stromal response (Fig. 3d, e) in *Kras*-driven mice with *Gpx4* depletion (KCG) or a high-iron diet. Accordingly, the mRNA expression of *Sox9*, *Krt19*, *Vim*, and *Mmp9* were downregulated by clophosome (Fig. 3f). Thus, accumulated macrophages appear to be necessary for high-iron diet- or *Gpx4* depletion-dependent pancreatic tumorigenesis.

### Oxidized nucleobase induces macrophage migration and activation ***via*** TMEM173

**Fig. 4:**
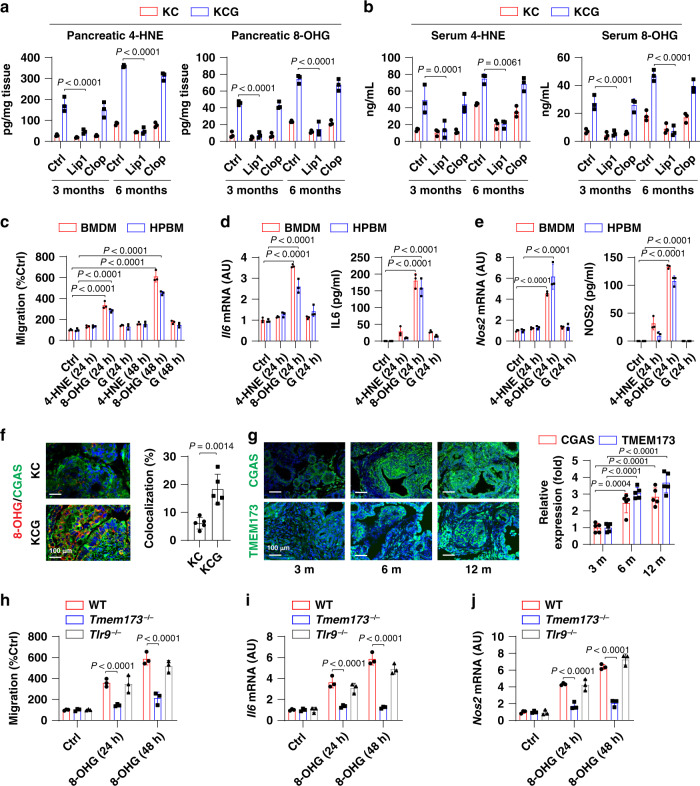
8-OHG induces macrophage migration and activity. **a**, **b** Levels of 4-HNE or 8-OHG in the pancreas (**a**) or serum (**b**) in *Pdx1-Cre;K-Ras*^*G12D/+*^ (KC) or *Pdx1-Cre;K-Ras*^*G12D/+*^*;Gpx4*^*−/−*^ (KCG) mice with or without liproxstatin-1 (Lip1) or clophosome (Clop) treatment (*n* = 3 mice/group; two-way ANOVA with Tukey’s multiple comparisons test). **c–e** Mouse bone marrow-derived macrophages (BMDMs) or human blood monocyte-derived macrophages (HPBMs) were treated with 4-HNE (500 ng/ml) or 8-OHG (500 ng/ml) or guanosine (“G”, 500 ng/ml) for 24 and 48 h. The cell migration (**c**) and mRNA levels or release of *Il6* (**d**) and *Nos2* (**e**) were assayed (*n* = 3; two-way ANOVA with Tukey’s multiple comparisons test). **f** Co-localization of 8-OHG DNA and CGAS in tumors of KC and KCG mice at 3 months of age (*n* = 5 mice/group; one-tailed *t* test). **g** Expression of CGAS and TMEM173 in KC mice at 3–12 months of age (*n* = 5 mice/group; two-way ANOVA with Tukey’s multiple comparisons test). **h**–**j** BMDMs from wild-type (WT), *Tmem173*^*−/−*^, or *Tlr9*^*−/−*^ mice were treated with 8-OHG (500 ng/ml) for 24 and 48 h. The cell migration (**h**) and mRNA levels of *Il6* (**i**) and *Nos2* (**j**) were assayed (*n* = 3; two-way ANOVA with Tukey’s multiple comparisons test). Data in **a**–**j** are presented as mean ± SD. Data are from two or three independent experiments.

Damage-associated molecular patterns (DAMPs) are endogenous molecules released by dead or dying cells that contribute to the regulation of immune response in the tumor microenvironment^22^. *Gpx4* depletion or a high-iron diet resulted in an increase in the production and release of oxidative DAMPs, including 4-hydroxynonenal (4-HNE, a lipid peroxidation product derived from oxidized ω-6 polyunsaturated fatty acids) and 8-hydroxy-2′-deoxyguanosine (8-OHG, a major product of oxidative DNA damage) in the pancreas (Fig. 4a and Supplementary 5a) or serum (Fig. 4b and Supplementary 5b). Similar to antibody-based enzyme-linked immunosorbent assay (ELISA) analysis, liquid chromatography-tandem mass spectrometry (LC-MS/MS) analysis observed that the level of 8-OHG in pancreatic DNA was increased under *Gpx4* depletion or a high-iron diet (Supplementary 5c). In contrast, liproxstatin-1 (but not clophosome) reduced *Gpx4* depletion- or high-iron diet-induced 4-HNE and 8-OHG production in the pancreas (Fig. 4a and Supplementary 5a) or serum (Fig. 4b and Supplementary 5b).

We next tested whether these oxidative damage-associated DAMPs can regulate macrophage migration and activation in vitro. Compared to 4-HNE, 8-OHG (500 ng/ml) significantly induced cell migration (Fig. 4c) and cytokine (e.g., *Il6* and *Nos2*) expression/release in primary mouse bone marrow-derived macrophages (BMDMs) or human blood monocyte-derived macrophages (HPBMs) (Fig. 4d, e). The control guanosine (500 ng/ml) failed to induce cell migration and cytokine expression/release in macrophages (Fig. 4c–e). In addition, 8-OHG at physiological concentrations (<5 ng/ml^23^) failed to induce the release of IL-6 in BMDM (Supplementary 5d). These results support the idea that oxidized nucleobases (e.g., 8-OHG), rather than oxidized lipids (e.g., 4-HNE), may promote macrophage migration and activation at pathological concentrations.

Given that 8-OHG is an oxidized DNA damage product^24^, we then tested whether TMEM173, a major DNA-sensing regulator in host cells^25,26^, regulates 8-OHG activity. The DNA sensor cyclic GMP-AMP synthase (CGAS) binds to host DNA to initiate a TMEM173-dependent reaction. Compared with control KC mice, the co-localization of 8-OHG DNA and CGAS in tumors from KCG mice increased (Fig. 4f), supporting the previous finding that 8-OHG is a direct ligand of CGAS^27^. During *Kras*-driven PDAC in mice, the expression of TMEM173 or CGAS increased in a time-dependent manner in the tumor microenvironment (Fig. 4g). In vitro, the depletion of *Tmem173* blocked 8-OHG-induced cell migration (Fig. 4h) and mRNA expression of *Il6* and *Nos2* (Fig. 4i, j) in BMDMs. In comparison, the depletion of CpG DNA receptor toll-like receptor 9 (*Tlr9*) showed no change in 8-OHG-induced cell migration (Fig. 4h) and cytokine expression (Fig. 4i, j) in BMDMs. Together, these data suggest that TMEM173, but not TLR9, is required for 8-OHG-induced macrophage migration and activation.

### TMEM173 facilitates pancreatic tumorigenesis

**Fig. 5:**
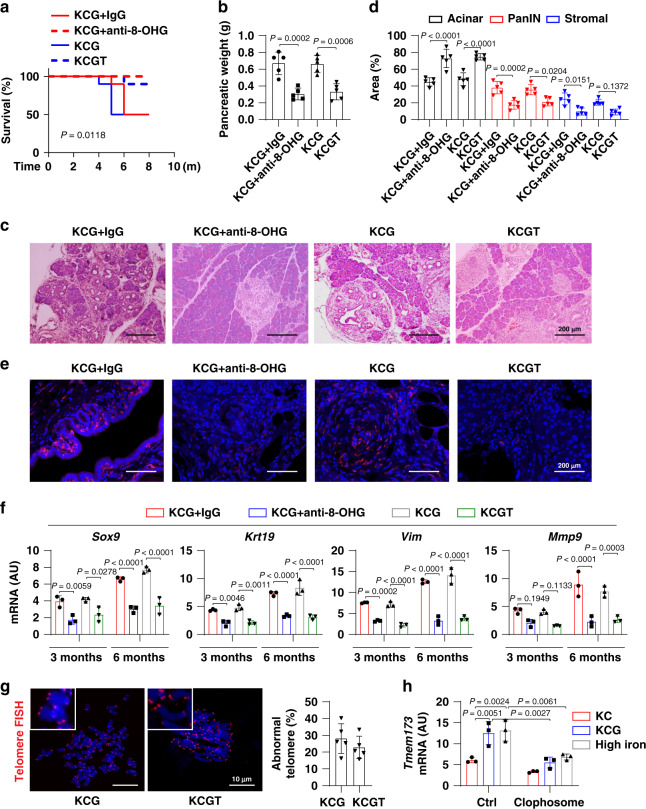
TMEM173 facilitates pancreatic tumorigenesis. **a** Survival of *Pdx1-Cre;Kras*^*G12D/+*^*;Gpx4*^*−/−*^ (KCG) or *Pdx1-Cre;Kras*^*G12D/+*^*; Gpx4*^*−/−*^*;Tmem173*^*−/−*^ (KCGT) mice with or without control IgG or anti-8-OHG antibody treatment (*n* = 10 mice/group; log-rank [Mantel–Cox] test). **b** Pancreatic weight of the indicated mice (6 months; *n* = 5 mice/group; two-way ANOVA with Tukey’s multiple comparisons test). **c** Representative pancreas histology of the indicated mice. **d** Percentages of histological structures in the pancreas of the indicated mice (*n* = 5 mice/group; two-way ANOVA with Tukey’s multiple comparisons test). **e** Representative images of immunofluorescence staining of macrophages (red) in pancreas in indicated mice at the age of 3 months. **f** Relative gene expression in the pancreas of the indicated mice (*n* = 3 mice/group; two-way ANOVA with Tukey’s multiple comparisons test). **g** Percentage of abnormal telomeres by FISH analysis in ductal cells from KCG and KCGT mice at 3 months of age (*n* = 5 mice/genotype). **h** mRNA expression of *Tmem173* in indicated mice at 3 months of age with or without clophosome treatment (*n* = 3 mice/group; two-way ANOVA with Tukey’s multiple comparisons test). Data in **b**, **d**, and **f**–**h** are presented as mean ± SD. Data are from two or three independent experiments.

To determine whether the TMEM173 pathway is required for pancreatic tumorigenesis, we first administered anti-8-OHG antibodies into *Kras*-driven mice with *Gpx4* depletion. Compared to control immunoglobulin G (IgG), anti-8-OHG antibody prolonged animal survival (Fig. 5a) and suppressed *Gpx4* depletion-mediated pancreatic weight gain (Fig. 5b) and rapid neoplastic progression with decreased PanIN formation (Fig. 5c, d), reduced stromal response (Fig. 5c, d), and decreased TAM infiltration (Fig. 5e). Similarly, the depletion of *Tmem173* (*Tmem173*^*−/−*^) protected against *Gpx4* depletion-induced animal death, neoplastic progression, and decreased TAM infiltration in *Kras*-driven mice (Fig. 5a–e). Blocking the 8-OHG-TMEM173 pathway also reduced *Gpx4* depletion-induced upregulation of *Sox9*, *Krt19*, *Vim*, and *Mmp9* in the pancreas (Fig. 5f). Moreover, the administration of anti-8-OHG antibodies or depletion of *Tmem173* protected against high-iron diet-induced animal death and neoplastic progression, and TAM infiltration in *Kras*-driven mice (Supplementary Fig. 6). Overtime 8-OHG accumulation may promote chromosomal instability, such as telomere abnormalities. Compared with control KCG mice, telomere fluorescent in situ hybridization (FISH) analysis showed that the depletion of *Tmem173* in KCGT mice did not significantly change telomere deficiency (Fig. 5g). Interestingly, macrophage depletion via clophosome reduced the increase in TMEM173 expression in KCG and high-iron diet mice (Fig. 5h), indicating that there is a feedback mechanism between macrophage infiltration, TMEM173 activation, and ferroptosis induction.

Collectively, these animal studies suggest that the activation of the 8-OHG-TMEM173 pathway contributes to pancreatic tumorigenesis in a telomere damage-independent manner.

### GPX4 is a prognostic marker in human PDAC

**Fig. 6:**
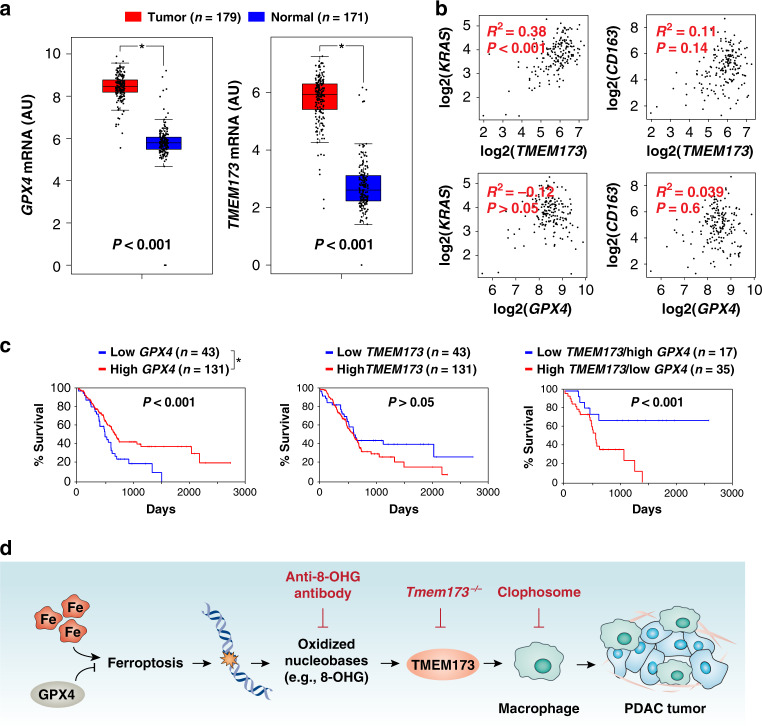
GPX4 is a prognostic marker in human PDAC. **a** Upregulation of *GPX4* and *TMEM173* gene expression within the tumor from PDAC patients compared to normal controls using datasets from The Cancer Genome Atlas (TCGA) (*P < 0.001; one-tailed *t* test). The data are presented as box-and-whisker plots. Boxes represent the median and the 25th and 75th percentiles. **b** Pearson correlation assay between *KRAS*, *CD163*, *TMEM173*, and *GPX4* gene expression in PDAC patient TCGA datasets. **c** Kaplan–Meier survival analysis with one-sided log-rank test of *GPX4* and *TMEM173* gene expression in PDAC patients using TCGA datasets. **d** Schematic depicting the role of high-iron diets or *Gpx4* depletion in *Kras*-driven PDAC. The induction of ferroptosis by either high-iron diets or *Gpx4* depletion promotes oxidized nucleobase (e.g., 8-OHG) release and thus activates the TMEM173-dependent DNA sensor pathway, which finally results in macrophage infiltration and activation during *Kras*-driven PDAC. Consequently, macrophage depletion or pharmacological and genetic inhibition of the 8-OHG-TMEM173 pathway suppresses ferroptosis-mediated pancreatic tumorigenesis.

Our animal studies indicate that GPX4 may play a tumor suppressor-like role, whereas *TMEM173* may have an oncogenic-like role in pancreatic tumorigenesis. To test this possibility, we carried out bioinformatics analyses using a publicly available gene expression dataset: The Cancer Genome Atlas (TCGA). Both *GPX4* and *TMEM173* mRNA expression was upregulated in the PDAC tumor group compared to the normal group (Fig. 6a). There was also a positive correlation between the mRNA expression of *KRAS* and *TMEM173* in patients with PDAC (Fig. 6b). In contrast, there was no significant correlation between the mRNA expression of *KRAS* and *GPX4* in patients with PDAC (Fig. 6b). Although macrophage depletion reduced TMEM173 expression in the tumor microenvironment in mice (Fig. 5h), there was no significant correlation between the mRNA expression of *GPX4* and *TMEM173* and macrophage marker *CD163* in patients with PDAC (Fig. 6b).

An overall survival assay further revealed that a high expression of *GPX4* was correlated with increased survival of PDAC patients (Fig. 6c). There was no significant correlation between the mRNA expression of *TMEM173* and overall survival of PDAC patients (Fig. 6c). In addition, the low expression of *GPX4* combined with the high expression of *TMEM173* increased the mortality of PDAC patients. These analyses indicate that GPX4 may be the main prognostic marker in human PDAC.

## Discussion

An oncogenic *Kras* mutation is the signature genetic event in the progression and growth of PDAC^2^. A recent study showed that deletion of a system x_C_-subunit, *Slc7a11*, induces ferroptosis and inhibits *Kras*-driven PDAC growth^28^. In this study, we identified a different mechanism, where ferroptotic damage may promote *Kras*-driven PDAC through the activation of the TMEM173-dependent DNA-sensing pathway in mice (Fig. 6d). These findings also support the need for careful consideration of immunological characteristics of tissue injury and cell death in studies of the tumor microenvironment with different contents^29,30^.

Since ferroptosis was described as an iron-dependent form of non-apoptotic cancer cell death in 2012, a growing number of studies of ferroptosis in cancer have boosted a perspective for the use of ferroptosis in cancer therapeutics^14^. Certain ferroptosis activators (e.g., erastin or RSL3) can be used to suppress tumor growth in vitro or in xenograft mouse models^10^. Compared with other regulators, GPX4 plays a central role in inhibiting ferroptosis under various conditions^31^. Our current study demonstrated that the conditional knockout of *Gpx4* in the pancreas accelerates a *Kras*-driven mouse model of PDAC, which can be reversed by a widely used ferroptosis inhibitor liproxstatin-1, although it may have potential off-target effects. Similarly, a high-iron diet also enhanced *Kras*-driven PDAC development and this process was also reversed by liproxstatin-1. Similar to oxidative stress^32,33^, ferroptosis-like damage may play a dual role in tumorigenesis and cancer therapy depending on the context, such as tumor type, stage, and genetic context^28,34–39^.

A marked infiltration of macrophages into the stromal compartment and the generation of a desmoplastic stromal reaction is a particular characteristic of PDAC^3^. Our study highlighted the importance of oxidative DNA damage in mediating *Kras*-driven tumorigenesis via infiltrated macrophages. Various types of oxidative DNA damage constitute a major threat to genetic integrity, and have thus been implicated in the pathogenesis of tumors^24,40^. *Gpx4* depletion or a high-iron diet increased the production and release of oxidized nucleobase (e.g., 8-OHG), which promotes subsequent macrophage accumulation and activation with the abnormal production of cytokines, especially IL-6 and NOS2. IL-6 and NOS2 are important players at all stages of PDAC and they correlate with poor survival^41,42^. Notably, the clophosome-mediated depletion of macrophages inhibited *Gpx4* depletion- or a high-iron diet-mediated pancreatic tumorigenesis, with reduced precursor lesion formation and stromal response, confirming the pathologic role of macrophages in PDAC^17,18^. Although the accumulation of 8-OHG occurs in various types of ferroptosis^43,44^, it remains to be investigated whether other types of oxidative DNA damage (such as alkylating damage^45^) are synergistically involved in tumor development mediated by ferroptotic damage.

We demonstrated that TMEM173, a regulator in inflammation and immune response, was responsible for 8-OHG-induced macrophage migration and activation. PDAC cells exhibit an enhanced dependence on iron for growth, whereas iron chelators can suppress pancreatic cancer growth in vitro and in vivo^46–48^. We found that a high-iron diet as well as *Gpx4* depletion accelerates *Kras*-driven PDAC progression. The TMEM173 pathway can recognize various DNA, including mitochondrial or genetic DNA, to induce type I interferon and cytokine production in both tumor and immune cells^25,26^. TMEM173 agonists have potent antitumor effects alone or in combination with other types of cancer treatments^49^. Conversely, the activation of TMEM173 by DNA damage or chromosomal instability promotes cancer growth and metastasis through the modulation of the tumor microenvironment^50^. Consistent with this finding, we demonstrated that *Gpx4* depletion- or a high-iron diet-mediated 8-OHG release could mediate macrophage infiltration and activation via the TMEM173 pathway. Each of these axes may represent a potential target for experimental therapeutic manipulation in PDAC. we demonstrated that the inhibition of the 8-OHG-TMEM173 pathway blocked *Kras*-driven PDAC initiation and development in mice. Our findings may reinforce the notion that cell death-mediated DAMP release as a component of the tumor microenvironment plays a potential pathological role in cancer development and progression.

In summary, the antioxidant GPX4 appears to be a key regulator of pancreatic tumorigenesis. Unraveling the complex metabolic regulation, immunological characteristics, and multiple diverse roles of oxidative damage may be pivotal in guiding the development of rational and novel PDAC prevention and therapies.

## Methods

### Reagents

Cerulean (#C9026) and l-arginine (#A5131) were obtained from Sigma-Aldrich. 8-OHG (#ab145594) was obtained from Abcam. 4-HNE (#2083) was obtained from BioVision. Clophosome (#F70101C-N) and control liposomes (#F70101-N) were obtained from FormuMax Scientific. Liproxstatin-1 (#S7699), guanosine (#S2439), celecoxib (#S1261), and PGE2 (#S3003) were obtained from Selleck Chemicals.

### Animal study

We conducted all animal care and experiments in accordance with the Association for Assessment and Accreditation of Laboratory Animal Care guidelines (http://www.aaalac.org) and with approval from our institutional animal care and use committee (Jilin University, UT Southwestern Medical Center, or Guangzhou Medical University). All mice were housed under a 12-h light–dark diurnal cycle with controlled temperature (20–25 °C) and relative humidity (40–60%). Food and water were available ad libitum. Experiments were carried out under pathogen-free conditions and the health status of mouse lines was routinely checked by veterinary staff. No wild animals were used in the study. Experiments were carried out with randomly chosen littermates of the same sex and matched by age and body weight. Animals were sacrificed at the indicated time by CO_2_ asphyxia, and blood samples and tissue were collected.

*Tlr9*^*−/−*^ mice on C57BL/6 background were kindly provided by Dr. Timothy R. Billiar (University of Pittsburgh). *Pdx1-Cre*, *Kras*^*G12D/+*^, and *Tmem173*^*−/−*^ mice on C57BL/6 background were received from the Jackson Laboratory. *Gpx4*^*flox/flox*^ mice on C57BL/6 background were obtained from Dr. Qitao Ran (University of Texas Health Science Center). These mice were crossed to generate indicated *Pdx1-Cre;Gpx4*^*−/−*^ (KO), *Pdx1-Cre;Kras*^*G12D/+*^*;Gpx4*^*−/−*^ (KCG), *Pdx1-Cre;Kras*^*G12D/+*^*;Tmem173*^*−/−*^ (KCT), or *Pdx1-Cre;Kras*^*G12D/+*^*;Gpx4*^*−/−*^*;Tmem173*^*−/−*^ (KCGT) animals. These mice were born at predicted Mendelian rates, such as KO (~50%), KCG (~25%), and KCT (~25%). Compared with the corresponding control group, the weight, blood glucose, and blood amylase of these Gpx4- and/or *Tmem173*-deficient animals had no significant differences at birth or 28 days of age (Supplementary Fig. 2). All mice used for pancreatitis (6–8 weeks old; male:female 1:1) and PDAC (1–10 months old; male: female: 1:1) models were matched for age and sex.

For l-arginine-induced pancreatitis, a sterile solution of l-arginine hydrochloride (8%) was prepared in normal saline and the pH was adjusted to 7.0. Mice received three-hourly intraperitoneal (i.p.) injections of l-arginine (3 g/kg), while controls were given saline^51^. For cerulein-induced pancreatitis, mice received seven-hourly i.p. injections of 50 μg/kg cerulein in sterile saline, while controls were given saline^52^.

In the feeding study, mice were fed modified AIN-93G rodent diet formulated to contain 50 p.p.m. iron (control diet; Envigo, #TD.130018) or 10,000 p.p.m. (1%) carbonyl iron (high-iron diet; Envigo, #TD.130015).

To study the effects of ferroptosis inhibitor on pancreatic tumorigenesis, 4–6 weeks old indicated that mice were randomly allocated into groups and injected i.p. with liproxstatin-1 (10 mg/kg) twice per week for 12 weeks. In addition, pretreatment with liproxstatin-1 (10 mg/kg) for 1 h was used in pancreatitis models.

To study the effects of 8-OHG inhibition on pancreatic tumorigenesis, 4–6 weeks old indicated that mice were randomly allocated into groups and injected i.p. with mouse monoclonal anti-8-OHG antibody (10 mg/kg; #GTX41980, RRID:AB_10732443, GeneTex) and control IgG2B (10 mg/kg; #MAB004, RRID:AB_357346, R&D Systems) per week for 12 weeks.

We did not exclude samples or animals. No statistical methods were used to predetermine sample sizes, but our sample sizes (*n* = 10 mice/group) for survival analysis are similar to those generally employed in the field^53^. All treatments were performed by technicians who were not blind, but not involved in sample measurement.

### Histological analysis

Pancreatic specimens were fixed with 10% buffered formalin, dehydrated in ethanol, embedded with paraffin, and stained with hematoxylin and eosin. The fraction of preserved acinar area was calculated as previously described^54^. Pancreatic ductal dysplasia was graded according to the established criteria^55,56^. Histological images were acquired using an EVOS FL Auto Cell Imaging System (Thermo Fisher Scientific).

### Immunofluorescence and immunohistochemistry analysis

For tissue immunofluorescence analysis, tissues were embedded in an optimum cutting temperature cryomedium (Sakura, Zoeterwoude) and subsequently cut into 5 μm sections. Tissue sections were stained with indicated primary antibodies (F4/80 [#ab6640, RRID:AB_1140040, Abcam, 1:200]; YM1 [#60130, RRID:AB_2868482, STEMCELL Technologies, 1:200]; cleaved caspase-3 [#9664, RRID:AB_2070042, Cell Signaling Technology, 1:200]; p-MLKL [#37333, RRID:AB_2799112, Cell Signaling Technology, 1:200]; CGAS [#sc-515777, RRID:AB_2734736, Santa Cruz Biotechnology, 1:50]; TMEM173 [#NBP2-24683, RRID:AB_2868483, Novus Biologicals, 1:100]; GPX4 [#MBS4380953, RRID:AB_2868488, MyBioSource, 1:100]; and 8-OHG [#NB600-1508, RRID:AB_787860, Novus Biologicals, 1:200]), followed by Cy3- (#A10521 [RRID:AB_1500665; 1:500] or #A10520 [RRID:AB_2534029; 1:500], Thermo Fisher Scientific) or Alexa Fluor 488-conjugated IgG (#A32766 [RRID:AB_2762823; 1:500] or #A32790 [RRID:AB_2762833; 1:500], Thermo Fisher Scientific). Nuclear morphology was analyzed with the fluorescent dye Hoechst 33342 (#H1399, Thermo Fisher Scientific). Immunohistochemistry in pancreatic cancer tissue array was performed using antibodies directed against Ki67 (#12202, RRID:AB_2620142, Cell Signaling Technology, 1:200) or GPX4 (#PA5-79321, RRID:AB_2746437, Thermo Fisher Scientific, 1:200). Telomere peptide nucleic acid FISH kits were obtained from Agilent (#K532611-8) and telomere morphological defects (such as chromosome fusion and concatenation, telomere signal-free end, and sister telomere fusion) were assayed as previously described^40^. Images were collected using a laser-scanning confocal microscope (Fluoview FV-1000; Olympus).

### Primary culture cells

BMDMs from *WT*, *Tmem173*^*−/−*^, and *Tlr9*^*−/−*^ mice were obtained using 30% L929 cell conditioned medium as a source of CSF2/granulocyte/macrophage colony-stimulating factor^57,58^. Primary HPBMs were obtained from STEMCELL Technologies (#70042). These cells were cultured in RPMI-1640 (#11875119, Thermo Fisher Scientific) supplemented with 10% heat-inactivated fetal bovine serum (#TMS-013-B, Millipore) and 1% penicillin and streptomycin (#15070-063, Thermo Fisher Scientific) at 37 °C, 95% humidity, and 5% CO_2_. All cells were tested to ensure that they were Mycoplasma-negative and were characterized by short tandem repeat profiling.

### ELISA analysis

The amylase (#ab102523, Abcam), myeloperoxidase level (#EMMPO, Thermo Fisher Scientific), trypsin (#ab102531, Abcam), 4-HNE (#STA-838, Cell Biolabs), 8-OHG (#K4160, BioVision), IL-6 (#EH2IL6 and #KMC0061, Thermo Fisher Scientific), and NOS2 (#ab253219 and #ab253217, Abcam) concentrations or activity in the samples were measured using ELISA kits.

### LC-MS/MS analysis

The DNA sample was hydrolyzed into nucleosides, and then 8-OHG in the sample was analyzed on a C18 chromatographic column using a binary gradient system composed of mobile phase A (98% H_2_O, 2% CH_3_CN (v/v), and 10 mM NH_4_CH_3_CO_2_) and mobile phase B (5% H_2_O, 95% CH_3_CN (v/v), and 10 mM NH_4_CH_3_CO_2_) as described previously^59^. An isocratic mode (92.5% A and 7.5% B) was used to achieve the desired sample separation at a flow rate of 0.25 ml/min. The column temperature was maintained at 40 °C. For each analysis, an aliquot of 2–10 µl of the hydrolyzed DNA sample (1 µg DNA/µl) was injected into the column. LC-MS/MS analyses were performed using a TSQ Quantum™ Access MAX Triple Quadrupole Mass Spectrometer (Thermo Fisher Scientific). Analysis by LC-MS/MS used selected-reaction monitoring mode with the mass transitions *m*/*z* 284 → *m*/*z* 168 for 8-OHG^60^.

### Q-PCR analysis

Total RNA was extracted and purified from cells or tissues using the RNeasy Plus Mini Kit (#74136, Qiagen). First-strand complementary DNA (cDNA) was synthesized from 1 µg of RNA using the iScript cDNA Synthesis Kit (#1708890, Bio-Rad). Briefly, 20 μl reactions were prepared by combining 4 μl iScript Select reaction mix, 2 μl gene-specific enhancer solution, 1 μl reverse transcriptase, 1 μl gene-specific assay pool (20×, 2 μM), and 12 μl RNA diluted in RNase-free water. Then cDNA from various cell samples was amplified by real-time quantitative polymerase chain reaction (Q-PCR) with primers using a CFX96 Touch Real-Time PCR Detection System (Bio-Rad). The expression of target genes was calculated using the ddCt method relative to the expression of a housekeeping gene, *Actb*/β-actin. The specific primers were listed in Supplementary Table 1. One limitation of Q-PCR on the whole pancreas was that it was difficult to separate the stroma-derived mRNA signal from that of the epithelial cells.

### Transwell migration assay

Transwell migration assays were performed with 500 μl cell culture medium with or without 4-HNE (500 ng/ml) or 8-OHG (500 ng/ml) in the lower chamber and 250 μl of macrophages (2 × 10^6^/ml) in the upper chamber/insert of a 24-well plate with a 3 μM pore-size membrane (#140627, Nunc) at 37 °C. Cells that migrated into the lower chamber were counted with a hemocytometer. Relative migrated cells of parental cells were set at 100%.

### Isolation of TAMs

Pancreas was minced into small fragments and incubated in collagenase solution (collagenase type I [45 U/ml], collagenase type II [15 U/ml], collagenase type III [45 U/ml], collagenase type IV [45 U/ml], elastase [0.08 U/ml], hyaluronidase [30 U/ml], and DNAse type I [25 U/ml] in RPMI-1640) on a shaker at 37 °C for 40 min. Dissociated cells were passed through a 70-μm cell strainer and washed twice in phosphate-buffered saline media. A MagniSort Mouse F4/80 Positive Selection Kit (#8802-6863-74, Thermo Fisher Scientific) was then used for the magnetic separation of F4/80+ cells in single-cell suspensions by positive selection.

### Bioinformatics analysis

The TCGA datasets, including gene expression and clinical outcomes data, were obtained from level 3 datasets at FireBrowse (http://gdac.broadinstitute.org, 2016 January). GEPIA (http://gepia.cancer-pku.cn/index.html), a newly developed interactive web server for analyzing the TCGA data, was used to separate the TCGA cohorts into groups with high/low expression of selected genes, which were then used for the prognostic signature validation based on the best cut-off values.

### Measuring glucose

Glucose concentration measurements were obtained from whole-blood samples using hand-held whole-blood glucose monitors (Bayer).

### Statistical analysis

GraphPad Prism 8.4.3 was used to collect and analyze data. Unpaired Student’s *t* tests were used to compare the means of two groups. A one- or two-way analysis of variance (ANOVA) with Tukey’s multiple comparisons test was used for comparison among the different groups. Log-rank test was used to compare differences in mortality rates between groups. The Pearson’s correlation was used to assess the relationship between gene expressions. A *P* value of <0.05 was considered statistically significant. We did not exclude samples or animals. No statistical methods were used to predetermine sample sizes, but our sample sizes are similar to those generally employed in the field^53^.

### Reporting summary

Further information on research design is available in the Nature Research Reporting Summary linked to this article.

## Supplementary information

Supplementary Information

Reporting Summary

## Data Availability

All the other data supporting the findings of this study are available within the article and its Supplementary information files and from the corresponding author upon reasonable request. Source data are provided with this paper.

## Source data

Source Data
