# Supplementary material for: Ferroptotic damage promotes pancreatic tumorigenesis through a TMEM173/STING-dependent DNA sensor pathway
Source: Nat Commun. 2020 Dec 11;11:6339. doi: 10.1038/s41467-020-20154-8 (PMC7732843; doi:10.1038/s41467-020-20154-8)
Supplement: Supplementary file 1 — Supplementary Information [file 41467_2020_20154_MOESM1_ESM.pdf]

Supplementary Information

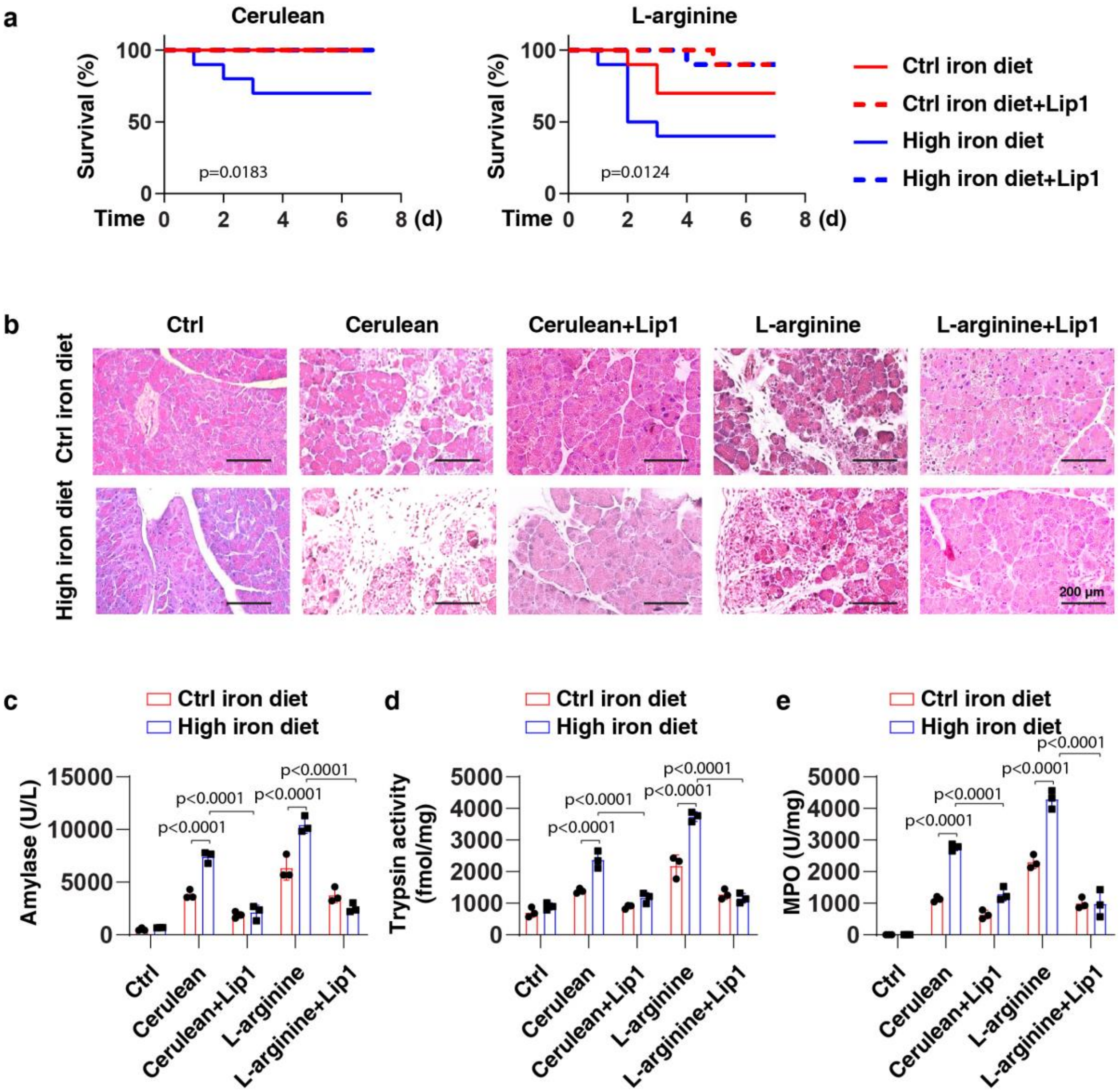

Supplementary Figure 1. A high-iron diet promotes experimental pancreatitis. (a) Survival of mice with control or high-iron diet in cerulean- or L-arginine-induced pancreatitis with or without liproxstatin-1 (Lip1) treatment (n = 10 mice/group; one-sided Log-rank [Mantel-Cox] test). (b-e) In parallel, pancreas histology (b), plasma amylase activity (c), pancreatic trypsin activity (d), and pancreatic myeloperoxidase (MPO) activity (e) at 24 hours were assayed (n = 3 mice/group; two way

ANOVA with Tukey's multiple comparisons test). Data in (c-e) are presented as mean ±SD. Data are from two or three independent experiments.

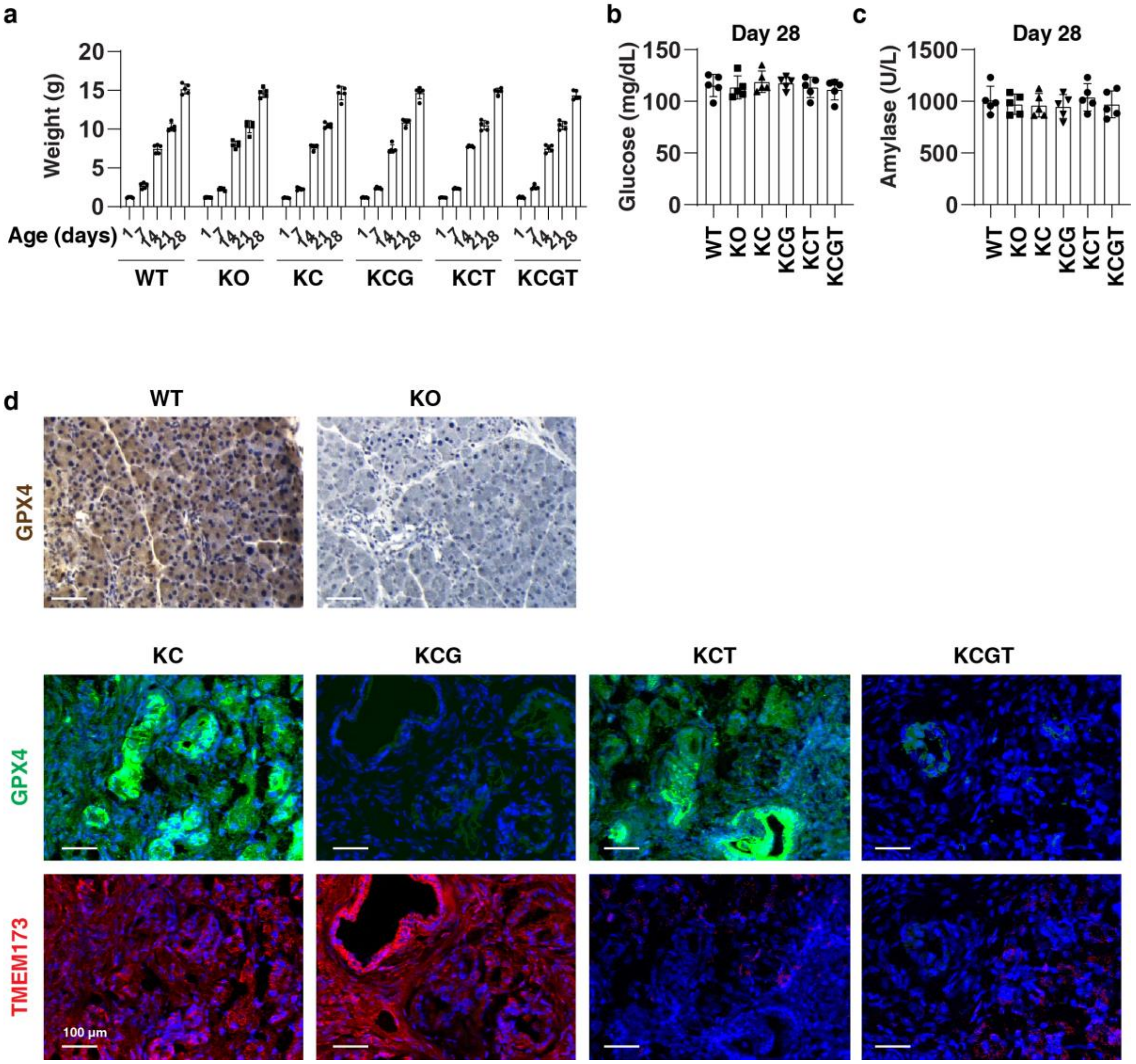

Supplementary Figure 2. Effect of GPX4 and/or TMEM173 deletion on pancreatic function. (a-c) The weight, blood glucose, and blood amylase of indicated mice. (d) IHC or IF staining of GPX4 and TMEM173 on indicated mice at the age of 3 months. Data in (a-c) are presented as mean ± SD (n = 5 mice/group). Data are from two independent experiments.

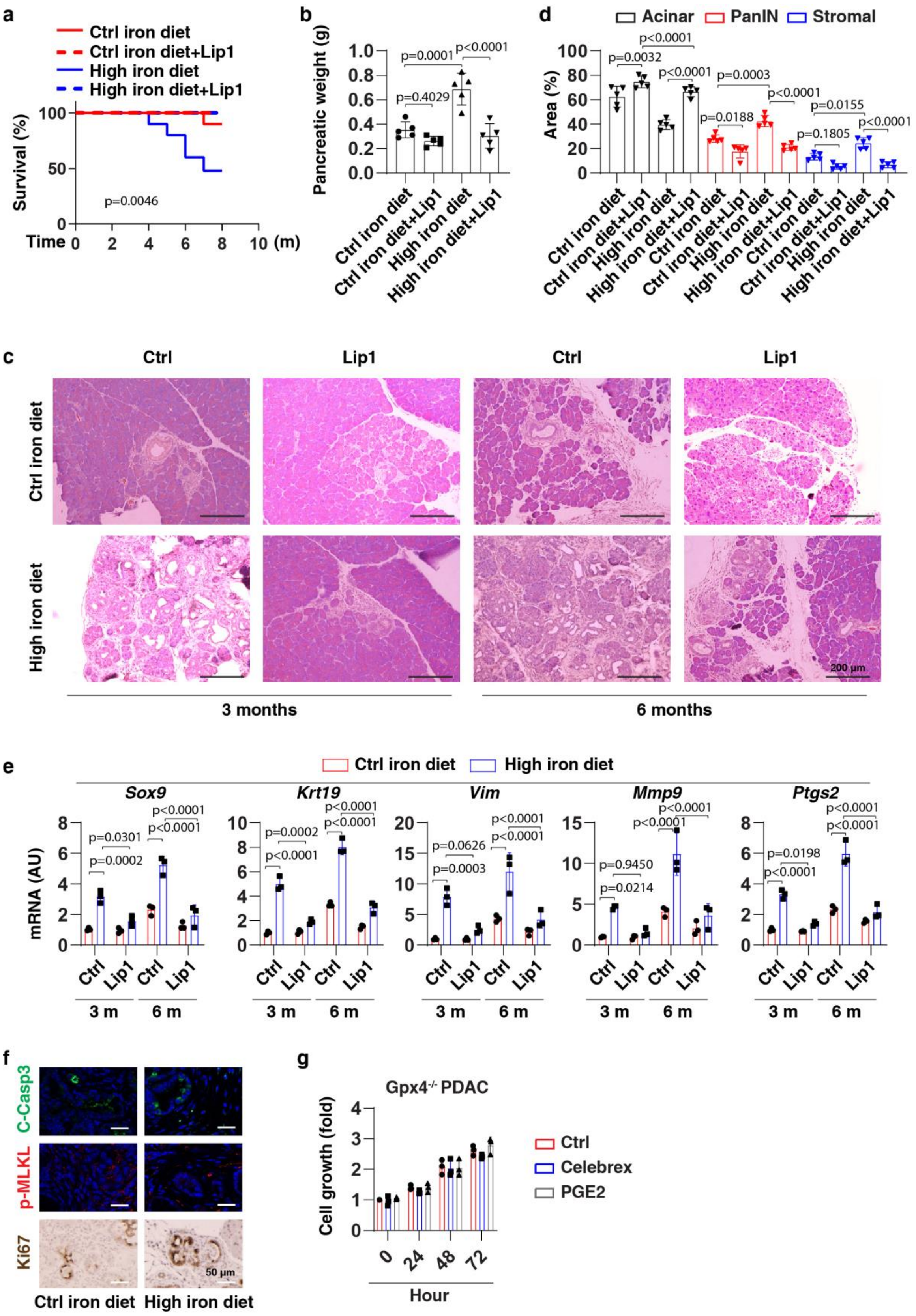

a
Ctrl iron diet
Ctrl iron diet+Lip1
High iron diet
High iron diet+Lip1
Survival (%)
p=0.0046
Time (m)
b
Pancreatic weight (g)
p=0.0001
p<0.0001
p=0.4029
d
Acinar
PanIN
Stromal
Area (%)
c
Ctrl
Lip1
Ctrl iron diet
High iron diet
200 μm
3 months
6 months
e
Ctrl iron diet
High iron diet
Sox9
Krt19
Vim
Mmp9
Ptgs2
mRNA (AU)
3 m
6 m
f
C-Casp3
p-MLKL
Ki67
50 μm
Ctrl iron diet
High iron diet
g
Gpx4-/- PDAC
Cell growth (fold)
Ctrl
Celebrex
PGE2
Hour

Supplementary Figure 3. A high-iron diet promotes *Kras*-driven pancreatic tumorigenesis. (a) Survival of *Pdx1-Cre;Kras*$^{G12D/+}$ (KC) mice with control or high-iron diet with or without liproxstatin-1 (Lip1) treatment (n = 10 mice/group; one-sided Log-rank [Mantel-Cox] test). (b) Pancreas weight of the indicated mice (6 months; n = 5 mice/group; one way ANOVA with Tukey's multiple comparisons test). (c) Representative pancreas histology of the indicated mice. (d) Percentages of histological structures in the pancreas of the indicated mice (6 months; n = 5 mice/group; two way ANOVA with Tukey's multiple comparisons test). (e, f) Relative gene or protein expression in the pancreas of the indicated mice (n = 3 mice/group; two way ANOVA with Tukey's multiple comparisons test). (g) Cell growth in primary Gpx4$^{-/-}$ PDAC cells in the absence or presence of celecoxib (50 nM) or PGE2 (5 ng/ml) for 24-72 hours. Data in (b, d, e, and g) are presented as mean ± SD. Data are from two or three independent experiments.

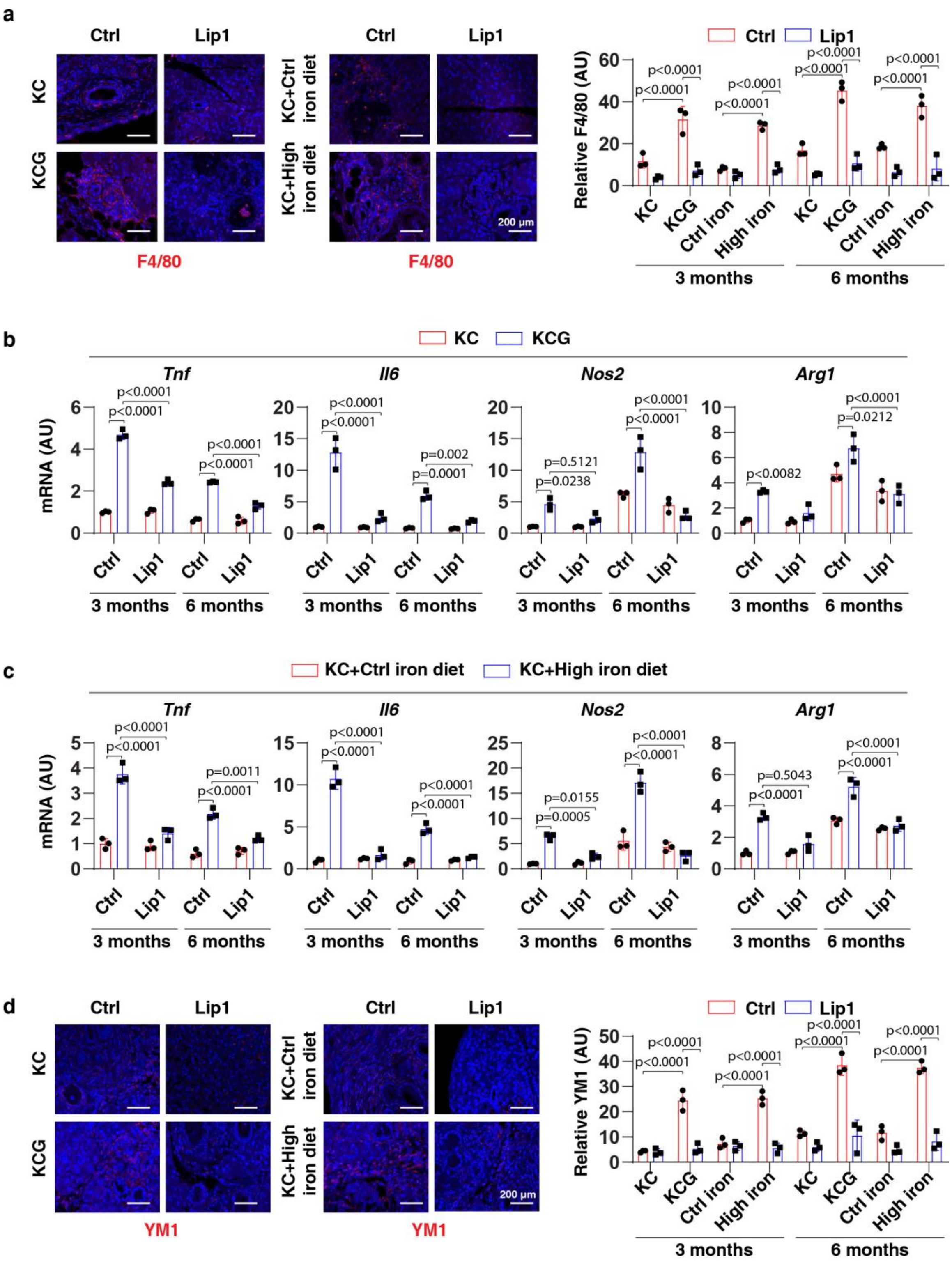

Supplementary Figure 4. ***Gpx4*** **depletion or a high-iron diet promotes macrophage infiltration and activation during pancreatic tumorigenesis.** (a) Immunofluorescence staining of macrophages in pancreas by anti-F4/80 antibody

(red) in indicated mice at the age of 3 and 6 months (n = 3 mice/group; two way ANOVA with Tukey's multiple comparisons test). (b, c) Relative gene expression in tumor-associated macrophages in indicated mice (n = 3 mice/group; two way ANOVA with Tukey's multiple comparisons test). (d) Immunofluorescence staining of YM1 (red) in pancreas in indicated mice at the age of 3 and 6 months (n = 3 mice/group; two way ANOVA with Tukey's multiple comparisons test). Data in (a-d) are presented as mean ± SD. Data are from two or three independent experiments.

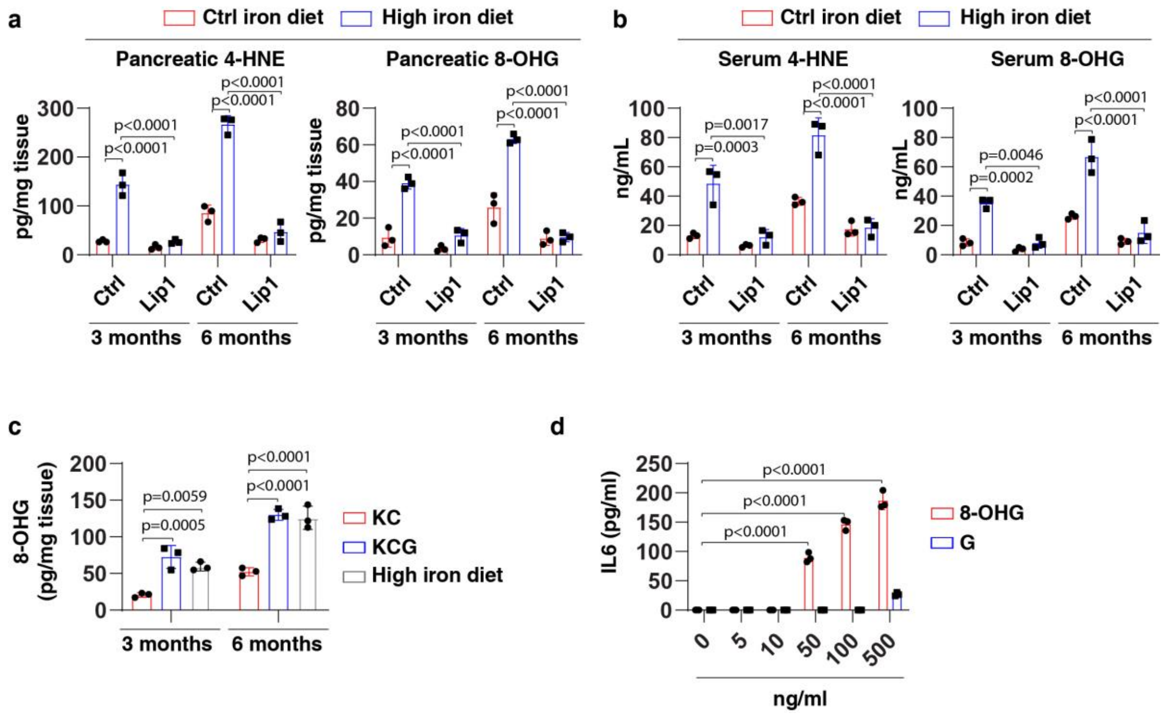

Supplementary Figure 5. A high-iron diet promotes 4-HNE and 8-OHG production and release. ELISA assay levels of 4-HNE or 8-OHG in the pancreas (a) or serum (b) in *Pdx1-Cre;Kras*$^{G12D/+}$ (KC) mice with control or high-iron diet (n = 3 mice/group; two way ANOVA with Tukey's multiple comparisons test). (c) LC-MS/MS assay levels of 8-OHG in the pancreas in KC, KCG, or KC mice with high-iron diet (n = 3 mice/group; two way ANOVA with Tukey's multiple comparisons test). (d) Mouse bone marrow-derived macrophages (BMDMs) were treated with 8-OHG (5-500 ng/ml) or guanosine ("G", 5-500 ng/ml) for 24 hours. The release of IL6 were assayed (n = 3; two way ANOVA with Tukey's multiple comparisons test). Data in (a-d) are presented as mean ± SD. Data are from two independent experiments.

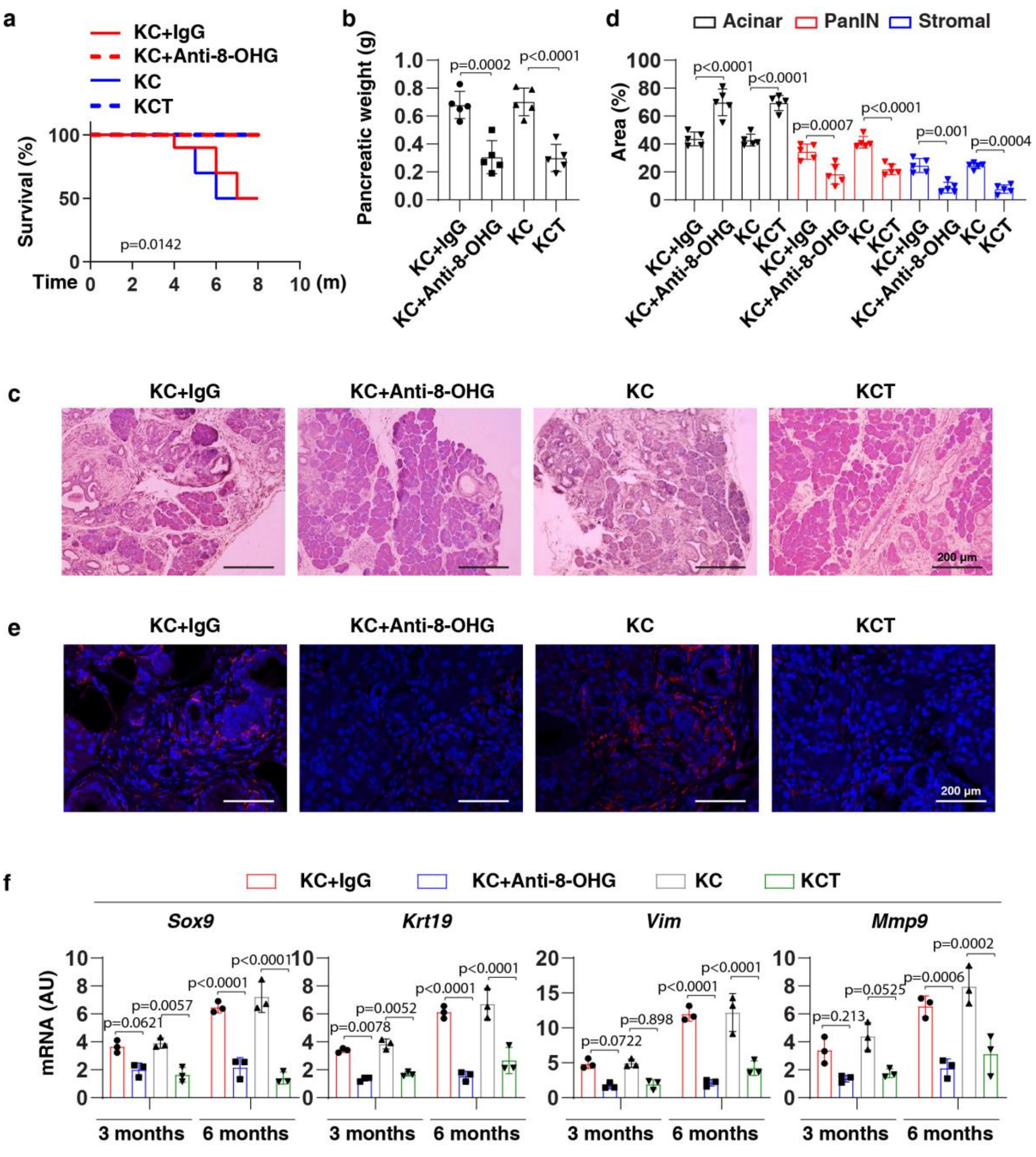

Supplementary Figure 6. TMEM173 facilitates pancreatic tumorigenesis. (a) Survival of *Pdx1-Cre;Kras*$^{G12D/+}$ (KC) or *Pdx1-Cre;Kras*$^{G12D/+}$*;Tmem173*$^{-/-}$ (KCT) mice with or without treatment of control IgG or anti-8-OHG antibody on high-iron diets (n =10 mice/group; one-sided Log-rank [Mantel-Cox] test). (b) Pancreas weight of the indicated mice (6 months; n = 5 mice/group; one way ANOVA with

Tukey's multiple comparisons test). (c) Representative pancreas histology of the indicated mice on high-iron diets. (d) Percentages of histological structures in the pancreas of the indicated mice at the age of 3 months on high-iron diets (n = 5 mice/group; two way ANOVA with Tukey's multiple comparisons test). (e) Representative images of immunofluorescence staining of macrophages (red) in pancreas of indicated mice at the age of 3 months on high-iron diets. (f) Relative gene expression in the pancreas of the indicated mice on high-iron diets (n = 3 mice/group; two way ANOVA with Tukey's multiple comparisons test). Data in (b, d, and f) are presented as mean ± SD. Data are from two or three independent experiments.

Supplementary Table 1

Primers used in this study

Gene	Forward	Reverse
mouse *Sox9*	5’-CACACGTCAAGCGACCCATGAA-3’	5’-TCTTCTCGCTCTCGTTCAGCAG-3’
mouse *Krt19*	5’-AATGGCGAGCTGGAGGTGAAGA-3’	5’- CTTGGAGTTGTCAATGGTGGCAC-3’
mouse *Vim*	5’-CGGAAAGTGGAATCCTTGCAGG-3’	5’-AGCAGTGAGGTCAGGCTTGGAA-3’
mouse *Mmp9*	5’-GCTGACTACGATAAGGACGGCA-3’	5’- TAGTGGTGCAGGCAGAGTAGGA-3’
mouse *Tnf*	5’- GGTGCCTATGTCTCAGCCTCTT-3’	5’-GCCATAGAACTGATGAGAGGGAG-3’
mouse *Il6*	5’-TACCACTTCACAAGTCGGAGGC-3’	5’- CTGCAAGTGCATCATCGTTGTTC-3’
mouse *Ptgs2*	5’- GCGACATACTCAAGCAGGAGCA-3’	5’-AGTGGTAACCGCTCAGGTGTTG-3’
human *IL6*	5’- AGACAGCCACTCACCTCTTCAG-3’	5’-TTCTGCCAGTGCCTCTTTGCTG-3’
mouse *Nos2*	5’-GAGACAGGGAAGTCTGAAGCAC-3’	5’- CCAGCAGTAGTTGCTCCTCTTC-3’
human *NOS2*	5’- GCTCTACACCTCCAATGTGACC-3’	5’-CTGCCGAGATTTGAGCCTCATG-3’
mouse *Arg1*	5’- CATTGGCTTGCGAGACGTAGAC-3’	5’- GCTGAAGGTCTCTTCCATCACC-3’
mouse *Actb*	5’-CTGTCCCTGTATGCCTCTG-3’	5’-ATGTCACGCACGATTTCC-3’
human *ACTB*	5’-AGCGAG CATCCCCCAAAGTT-3’	5’-AGGGCA CGAAGGCTCATCATT-3’
