# Supplementary material for: Ferroptotic damage promotes pancreatic tumorigenesis through a TMEM173/STING-dependent DNA sensor pathway
Source: Nat Commun. 2020 Dec 11;11:6339. doi: 10.1038/s41467-020-20154-8 (PMC7732843; doi:10.1038/s41467-020-20154-8)
Supplement: Supplementary file 2 — Reporting Summary [file 41467_2020_20154_MOESM2_ESM.pdf]

nature research

Corresponding author(s): Daolin Tang; Lulu Bai

Last updated by author(s): Nov 1, 2020

Reporting Summary

Nature Research wishes to improve the reproducibility of the work that we publish. This form provides structure for consistency and transparency in reporting. For further information on Nature Research policies, see Authors & Referees and the Editorial Policy Checklist.

Statistics

For all statistical analyses, confirm that the following items are present in the figure legend, table legend, main text, or Methods section.

n/a	Confirmed	
☐	☒	The exact sample size (*n*) for each experimental group/condition, given as a discrete number and unit of measurement
☐	☒	A statement on whether measurements were taken from distinct samples or whether the same sample was measured repeatedly
☐	☒	The statistical test(s) used AND whether they are one- or two-sided<br>*Only common tests should be described solely by name; describe more complex techniques in the Methods section.*
☒	☐	A description of all covariates tested
☒	☐	A description of any assumptions or corrections, such as tests of normality and adjustment for multiple comparisons
☐	☒	A full description of the statistical parameters including central tendency (e.g. means) or other basic estimates (e.g. regression coefficient) AND variation (e.g. standard deviation) or associated estimates of uncertainty (e.g. confidence intervals)
☐	☒	For null hypothesis testing, the test statistic (e.g. *F*, *t*, *r*) with confidence intervals, effect sizes, degrees of freedom and *P* value noted<br>*Give P values as exact values whenever suitable.*
☒	☐	For Bayesian analysis, information on the choice of priors and Markov chain Monte Carlo settings
☒	☐	For hierarchical and complex designs, identification of the appropriate level for tests and full reporting of outcomes
☐	☒	Estimates of effect sizes (e.g. Cohen's *d*, Pearson's *r*), indicating how they were calculated

Our web collection on statistics for biologists contains articles on many of the points above.

Software and code

Policy information about availability of computer code

Data collection	GraphPad Prism 8.4.3 was used to collect and analyze data
Data analysis	GraphPad Prism 8.4.3 was used to collect and analyze data

For manuscripts utilizing custom algorithms or software that are central to the research but not yet described in published literature, software must be made available to editors/reviewers. We strongly encourage code deposition in a community repository (e.g. GitHub). See the Nature Research guidelines for submitting code & software for further information.

Data

Policy information about availability of data

All manuscripts must include a data availability statement. This statement should provide the following information, where applicable:

- Accession codes, unique identifiers, or web links for publicly available datasets
- A list of figures that have associated raw data
- A description of any restrictions on data availability

All the other data supporting the findings of this study are available within the article and its supplementary information files and from the corresponding author upon reasonable request. Source data are provided with this paper.

Field-specific reporting

Please select the one below that is the best fit for your research. If you are not sure, read the appropriate sections before making your selection.

☒ Life sciences ☐ Behavioural & social sciences ☐ Ecological, evolutionary & environmental sciences

Life sciences study design

All studies must disclose on these points even when the disclosure is negative.

Sample size	No statistical methods were used to predetermine sample sizes, but our sample sizes (n=10 mice/group) for survival assay are similar to those generally employed in the field (EBioMedicine 48, 161-168 (2019)).
Data exclusions	No data was excluded.
Replication	Replicates in all assays were confirmatory and the extent described within the text and shown in the main figures.
Randomization	Animal experiments were carried out with randomly chosen littermates of the same sex and matched by age and body weight. For in vitro studies, all cells from multiple dishes were combined and then plated into wells that were treated with various drugs. Thus, all treatment groups came from the same cell stock. Biological independent experiments were performed on independent aliquots of cells thawed from the liquid nitrogen freezer.
Blinding	Animal treatments were performed by technicians who were not blind, but not involved in sample measurement. All in vitro experiments were not blind, because postdoctoral fellows had their own independent projects, and it is impossible for others to replace them to treat cells and analyze samples.

Reporting for specific materials, systems and methods

We require information from authors about some types of materials, experimental systems and methods used in many studies. Here, indicate whether each material, system or method listed is relevant to your study. If you are not sure if a list item applies to your research, read the appropriate section before selecting a response.

Materials & experimental systems

n/a	Involved in the study
☐	☒ Antibodies
☐	☒ Eukaryotic cell lines
☒	☐ Palaeontology
☐	☒ Animals and other organisms
☒	☐ Human research participants
☒	☐ Clinical data

Methods

n/a	Involved in the study
☒	☐ ChIP-seq
☒	☐ Flow cytometry
☒	☐ MRI-based neuroimaging

Antibodies

Antibodies used	All antibodies were commercial antibodies and were listed in Materials and methods: F4/80 [#ab6640, RRID:AB_1140040, Abcam, 1:200]; YM1 [#60130, RRID:AB_2868482, STEMCELL Technologies, 1:200]; cleaved caspase-3 [#9664, RRID:AB_2070042, Cell Signaling Technology, 1:200]; p-MLKL [#37333, RRID:AB_2799112, Cell Signaling Technology, 1:200]; CGAS [#sc-515777, RRID:AB_2734736, Santa Cruz Biotechnology, 1:50]; TMEM173 [#NBP2-24683, RRID:AB_2868483, Novus Biologicals, 1:100]; GPX4 [#MBS4380953, RRID:AB_2868488, MyBioSource, 1:100]; and 8-OHG [#NB600-1508, RRID:AB_787860, Novus Biologicals, 1:200]; Ki67 (#12202, RRID:AB_2620142, Cell Signaling Technology, 1:200) or GPX4 (#PA5-79321, RRID:AB_2746437, Thermo Fisher Scientific, 1:200). Cy3- (#A10521 [RRID:AB_1500665; 1:500] or #A10520 [RRID:AB_2534029; 1:500], Thermo Fisher Scientific) or Alexa Fluor 488-conjugated IgG (#A32766 [RRID:AB_2762823; 1:500] or #A32790 [RRID:AB_2762833; 1:500], Thermo Fisher Scientific). For animal experiments, anti-8-OHG antibody (10 mg/kg; #GTX41980, RRID:AB_10732443, GeneTex) and control IgG2B (10 mg/kg; #MAB004, RRID:AB_357346, R&D Systems) were used.
Validation	Below are validation statements from manufacturers as well as validation performed in-house and by other investigators.<br>F4/80 [#ab6640, Rat monoclonal [CI:A3-1]; IF, https://www.abcam.com/f480-antibody-cia3-1-macrophage-marker-ab6640.html];<br>YM1 [#60130, Rabbit polyclonal; IF, https://www.stemcell.com/anti-ym1-antibody-polyclonal.html]; cleaved caspase-3 [#9664, Rabbit mAb; IF, https://www.cellsignal.com/products/primary-antibodies/cleaved-caspase-3-asp175-5a1e-rabbit-mab/9664?Ntk=Products&Ntt=9664];<br>p-MLKL [#37333, Rabbit mAb IF, https://www.cellsignal.com/products/antibody-conjugates/phospho-mlkl-ser345-d6e3g-rabbit-mab/37333];<br>CGAS [#sc-515777, Mouse monoclonal [D-9]; IF,https://www.scbt.com/zh/p/cgas-antibody-d-9]; TMEM173 [#NBP2-24683, Rabbit polyclonal, IF, https://www.novusbio.com/products/sting-tmem173-antibody_nbp2-24683];<br>GPX4 [#MBS4380953, Mouse monoclonal [LHM2], IF, https://www.mybiosource.com/monoclonal-human-mouse-rat-antibody/gpx4-mcsp/4380953];<br>8-OHG [#NB600-1508, Goat polyclonal, IF, https://www.novusbio.com/products/8-ohdg-antibody_nb600-1508]; Ki67 (#12202, Rabbit mAb, IF, https://www.cellsignal.com/products/primary-antibodies/ki-67-d3b5-rabbit-mab-mouse-preferred-ihc-formulated/12202)<br>GPX4 (#PA5-79321, Rabbit polyclonal, IF, https://www.thermofisher.com/antibody/product/GPX4-Antibody-Polyclonal/PA5-79321)

Cy3-conjugated IgG (#A10521 [Goat anti-Mouse IgG (H+L); IF, https://www.thermofisher.com/antibody/product/Goat-anti-Mouse-IgG-H-L-Cross-Adsorbed-Secondary-Antibody-Polyclonal/A10521] or #A10520 [Goat anti-Rabbit IgG (H+L); IF,https://www.thermofisher.com/antibody/product/Goat-anti-Rabbit-IgG-H-L-Cross-Adsorbed-Secondary-Antibody-Polyclonal/A10520], Thermo Fisher Scientific)
Alexa Fluor 488-conjugated IgG (#A32766 [Donkey anti-Mouse IgG (H+L); IF; https://www.citeab.com/antibodies/6232905-a32766-donkey-anti-mouse-igg-h-l-highly-cross-adso; https://www.thermofisher.com/antibody/product/Donkey-anti-Mouse-IgG-H-L-Highly-Cross-Adsorbed-Secondary-Antibody-Polyclonal/A32766] or #A32790 [Donkey anti-Rabbit IgG (H+L); IF; https://www.thermofisher.com/antibody/product/Donkey-anti-Rabbit-IgG-H-L-Highly-Cross-Adsorbed-Secondary-Antibody-Polyclonal/A32790], Thermo Fisher Scientific).
anti-8-OHG antibody (Mouse monoclonal [15A3]; neutralizing, #GTX41980, https://www.genetex.com/Product/Detail/8-Hydroxyguanosine-antibody-15A3/GTX41980)
Mouse IgG2B Isotype Control (#MAB004, neutralizing, https://www.rndsystems.com/cn/products/mouse-igg2b-isotype-control_mab004)

Eukaryotic cell lines

Policy information about cell lines

Cell line source(s)	Bone marrow-derived macrophages (BMDMs) from WT, Tmem173-/-, and Tlr9-/- mice (male, 6-8 weeks) were obtained using 30% L929-cell conditioned medium as a source of CSF2/granulocyte/macrophage colony stimulating factor. Primary human blood monocyte-derived macrophages (HPBMs) were obtained from STEMCELL Technologies (#70042).
Authentication	All cells used were authenticated using STR profiling.
Mycoplasma contamination	Mycoplasma testing was negative.
Commonly misidentified lines (See ICLAC register)	No commonly misidentified cell lines were used in the study.

Animals and other organisms

Policy information about studies involving animals; ARRIVE guidelines recommended for reporting animal research

Laboratory animals	Tlr9-/- mice on C57BL/6 background were kindly provided by Dr. Timothy R. Billiar (University of Pittsburgh). Pdx-1-Cre, KrasG12D/+, and Tmem173-/- mice on C57BL/6 background were received from the Jackson Laboratory. Gpx4flox/flox mice on C57BL/6 background were obtained from Dr. Qitao Ran (University of Texas Health Science Center). These mice were crossed to generate indicated Pdx1-Cre;Gpx4-/- (KO), Pdx1-Cre;KrasG12D/+;Gpx4-/- (KCG), Pdx1-Cre;KrasG12D/+;Tmem173-/- (KCT), or Pdx1-Cre;KrasG12D/+;Gpx4-/-;Tmem173-/- (KCGT) animals. All mice used for pancreatitis (6-8 weeks old; male: female: 1:1) and PDAC (1-10 months old; male: female: 1:1) models were matched for age and sex.
Wild animals	No wild animals were used in the study.
Field-collected samples	No field collected samples were used in the study.
Ethics oversight	We conducted all animal care and experimentation in accordance with the Association for Assessment and Accreditation of Laboratory Animal Care guidelines (http://www.aaalac.org) and with approval from institutional animal care and use committees (Jilin University, UT Southwestern Medical Center, or Guangzhou Medical University) .

Note that full information on the approval of the study protocol must also be provided in the manuscript.
